# Genetic analyses of aplastic anemia and idiopathic pulmonary fibrosis patients with short telomeres, possible implication of DNA-repair genes

**DOI:** 10.1186/s13023-019-1046-0

**Published:** 2019-04-17

**Authors:** Elena G. Arias-Salgado, Eva Galvez, Lurdes Planas-Cerezales, Laura Pintado-Berninches, Elena Vallespin, Pilar Martinez, Jaime Carrillo, Laura Iarriccio, Anna Ruiz-Llobet, Albert Catalá, Isabel Badell-Serra, Luis I. Gonzalez-Granado, Andrea Martín-Nalda, Mónica Martínez-Gallo, Ana Galera-Miñarro, Carmen Rodríguez-Vigil, Mariana Bastos-Oreiro, Guiomar Perez de Nanclares, Virginia Leiro-Fernández, Maria-Luz Uria, Cristina Diaz-Heredia, Claudia Valenzuela, Sara Martín, Belén López-Muñiz, Pablo Lapunzina, Julian Sevilla, María Molina-Molina, Rosario Perona, Leandro Sastre

**Affiliations:** 1 Instituto de Investigaciones Biomedicas CSIC/UAM, IDIPaz, Arturo Duperier, 4, 28029 Madrid, Spain; 2 Advanced Medical Projects, Madrid, Spain; 3 Hospital Niño Jesús, Hematología y Hemoterapia, Madrid, Spain; 4 ILD Unit Pneumology Department, University Hospital of Bellvitge, IDIBELL, University of Barcelona, Barcelona, Spain; 5 Institute of Medical and Molecular Genetics (INGEMM), Hospital Universitario La Paz, Madrid, Spain; 6 Pediatric Hematology and Oncology Department, Hospital Sant Joan de Déu, University of Barcelona, Barcelona, Spain; 7 Institut de Recerca Pediàtrica Hospital Sant Joan de Déu (IRP-HSJD), Esplugues de Llobregat, Barcelona, Spain; 8 Hospital de la Santa Creu i Sant Pau, Barcelona, Spain; 9 Hospital 12 de Octubre, Madrid, Spain; 10 Immunology Division, Pediatric Infectious Diseases and Immunodeficiencies Unit, Hospital Universitari Vall d’Hebron (HUVH), Vall d’Hebron Research Institute (VHIR), Department of Cell Biology, Physiology and Immunology, Autonomous University of Barcelona (UAB), Barcelona, Spain; 11 Hospital Universitario Virgen de la Arrixaca, Murcia, Spain; 12 Hospital Miguel Servet, Zaragoza, Spain; 13 Hospital Universitario Gregorio Marañon, IiSGM, Madrid, Spain; 14 Molecular (Epi)Genetics Laboratory, BioAraba National Health Institute, OSI Araba University Hospital, Vitoria-Gasteiz, Spain; 15 Pneumology Department, Hospital Álvaro Cunqueiro, Complexo Hospitalario Universitario de Vigo, NeumoVigoI+i Research Group, Vigo Biomedical Research Institute (IBIV), Barcelona, Spain; 16 Hospital de La Princesa, Madrid, Spain; 17 Hospital Infanta Leonor, Madrid, Spain; 18 CIBER de enfermedades raras (CIBERER), Madrid, Spain; 19 CIBER of Respiratory diseases (CIBERES), Barcelona, Spain

**Keywords:** Telomere, Dyskeratosis congenita, Pulmonary fibrosis, Aplastic anemia, DNA repair, Telomeropathies

## Abstract

**Background:**

Telomeres are nucleoprotein structures present at the terminal region of the chromosomes. Mutations in genes coding for proteins involved in telomere maintenance are causative of a number of disorders known as telomeropathies. The genetic origin of these diseases is heterogeneous and has not been determined for a significant proportion of patients.

**Methods:**

This article describes the genetic characterization of a cohort of patients. Telomere length was determined by Southern blot and quantitative PCR. Nucleotide variants were analyzed either by high-resolution melting analysis and Sanger sequencing of selected exons or by massive sequencing of a panel of genes.

**Results:**

Forty-seven patients with telomere length below the 10% of normal population, affected with three telomeropathies: dyskeratosis congenita (4), aplastic anemia (22) or pulmonary fibrosis (21) were analyzed. Eighteen of these patients presented known pathogenic or novel possibly pathogenic variants in the telomere-related genes *TERT*, *TERC*, *RTEL1*, *CTC1* and *ACD*. In addition, the analyses of a panel of 188 genes related to haematological disorders indicated that a relevant proportion of the patients (up to 35%) presented rare variants in genes related to DNA repair or in genes coding for proteins involved in the resolution of complex DNA structures, that participate in telomere replication. Mutations in some of these genes are causative of several syndromes previously associated to telomere shortening.

**Conclusion:**

Novel variants in telomere, DNA repair and replication genes are described that might indicate the contribution of variants in these genes to the development of telomeropathies. Patients carrying variants in telomere-related genes presented worse evolution after diagnosis than the rest of patients analyzed.

**Electronic supplementary material:**

The online version of this article (10.1186/s13023-019-1046-0) contains supplementary material, which is available to authorized users.

## Background

Chromosome ends are protected by nucleoprotein structures, the telomeres. In humans, telomere DNA is composed by multiple repetitions of the TTAGGG hexanucleotide [1]. The 3′ end of telomeres is formed by a G-rich single stranded extension that invades the upstream double-stranded region to form telomeric loops (T-, D-loop). Telomere DNA is bound by a protein complex named shelterin [2] and this nucleo-protein structure protects chromosome ends from degradation and is critical for genome stability (recently reviewed in [3]).

Telomeres cannot be completely synthesized during DNA replication, which is known as the end-replication problem and results in progressive shortening of the telomeres as cells proliferate. In most organisms, telomere DNA is elongated after each round of DNA replication by a specialized complex with reverse transcriptase activity known as telomerase [4]. The enzymatic activity of the complex is provided by the TERT (telomerase reverse transcriptase) protein while the RNA component TR (telomerase RNA, encoded by *TERC*) is used as template. Essential components of the complex are also DKC (Dyskeratosis congenita), NOP10, NHP2 and GAR1 proteins, which bind and stabilize TR and are required for telomerase complex assembly [5].

Telomere replication, elongation and protection are impaired by the strong secondary and tertiary structure of their DNA [6]. The high content of Guanines of the telomeres makes them prone to form secondary structures such as G-quadruplexes. In addition, the T- and D-loops impair the access of the replicative machinery. These structures are solved by helicases and structure-specific endonucleases.

In the absence of a functional telomerase complex, telomeres are progressively shortened. One of the main functions of telomere structure is to avoid the recognition of telomeres as damaged DNA which is achieved by inhibitory interactions between shelterin components and DNA-repair proteins [7]. When several telomeres reach critically short length the structure of the nucleoprotein complex can no longer be maintained. In that case, telomeres are recognized as damaged DNA and a response is triggered that can result in cell-cycle arrest, cell apoptosis or senescence [8].

The presence of functional mutations in the genes coding for proteins of the shelterin and telomerase complexes and auxiliary proteins causes a number of diseases named telomeropathies or telomere biology disorders that may affect different organs [9, 10]. Telomeropathies are very heterogeneous diseases depending on the gene mutated and the specific mutations, their penetrance and the existence of anticipation effects. Therefore, patients with the same mutation can present different manifestations. Some patients present severe symptoms at an early age such as those of dyskeratosis congenita (DC) or the related Hoyeraal-Hreidarsson, Resvesz and Coats plus syndromes [11]. Other diseases may appear later such as Aplastic Anemia, (AA) (20–30 years) [12] or pulmonary fibrosis (40–60 years) (PF) [13]. Mutations in 13 different genes have been identified as causative of these diseases but a large number of patients remain genetically undiagnosed [14].

Variants in the gene coding for one helicase, RTEL1 (OMIM 608833) are also present in patients with telomeropathies [15]. Others helicases and nucleases, like SLX4, BLM, WRN and RecQL4, have been shown to participate in telomere preservation [16]. Actually, mutations in some of these genes also result in telomere shortening [16, 17]. The term secondary telomeropathies has been recently proposed for diseases caused by mutations in this group of genes [18].

The reduced number of patients of these rare diseases and the number of putative candidate genes makes their molecular diagnosis challenging. In this article, the molecular analysis of 47 patients treated in Spanish hospitals was performed. Twenty-six of them were diagnosed of DC or AA and twenty-one of PF. The results obtained identified possibly pathogenic variants at the telomere-associated genes *TERT*, *TERC*, *ACD*, *CTC1* and *RTEL1* in heterozygosis. In addition, a significant proportion of patients presented rare variants in genes coding for proteins involved in DNA repair and in the resolution of complex DNA structures.

## Methods

### Patients

Patients with severe telomere shortening (percentile < 10) who presented dyskeratosis congenita (DC), aplastic anemia (AA) or pulmonary fibrosis (PF) (sporadic and familial forms) were included. These patients were diagnosed, treated and followed in Spanish reference health care provider centres for these rare diseases. DC was diagnosed in patients with the characteristic mucocutaneous symptoms, nail dystrophy, abnormal skin pigmentation, oral leukoplakia and bone marrow failure. The diagnosis of AA was based on bone marrow and blood cells counts. The diagnosis of PF was established in accordance to international guidelines [19]. The more relevant characteristics of the patients are summarized in Additional file 1: Table S1. All samples were collected after obtaining informed consent for genetic analysis, including for research. Clinical and demographic data were collected.

### Genetic analyses

The existence of sequence variants in the *TERT*, *TERC* and *DKC1* genes was determined in six patients by PCR amplification of exons, high resolution melting analyses (HRM) and sequencing of candidate exons as previously described [20]. Later on, genetic analysis of 41 patients was made by massive parallel sequencing using a panel of genes related to haematological disorders, as shown in Additional file 1: Table S2 (MBFSv1.1 panel). Detailed protocols are presented in Additional file 1: Supplemental Material.

### Telomere length determination

Telomere length was determined by two different methods, Southern blots of enzymatically digested DNA (Additional file 1: Figure S1) and quantitative PCR. Both methods have been previously used for determination of telomere length from patients with inherited bone marrow failure [21]. Detailed protocols are presented in Additional file 1: Supplemental Material.

### Ethical statement

All the participants in this study provided written informed consent for genetic analysis. The investigation was conducted in accordance with the ethical standards of the Declaration of Helsinki and the guidelines of the concerned hospitals.

## Results

### Single nucleotide variants in genes related to telomere biology

The possible genetic bases of the diseases were studied in patients that presented symptoms related to dyskeratosis congenita (DC), either mucocutaneous symptoms or, more often, aplastic anemia (AA) or sporadic or familial cases of pulmonary fibrosis (PF). Patients with these diseases present significant telomere shortening. According to previous reports [12, 22], patients with telomere length below the 1 % of the age-mated population in the case of DC and < 10% in AA and PF where included in this study. Beside clinical symptoms, DC/AA and PF patients differed at the age of presentation of the disease, 12.2 + 12.2 years for DC/AA and 58.3 + 10.2 years for PF (Additional file 1: Table S1).

First analyses were made in six patients by PCR amplification of exons of the *DKC*, *TERT* and *TERC* genes using the previously described High-resolution melting and DNA sequencing method [20] (Patients indicated by an asterisk in Tables 1, 4 and Additional file 1: Table S1). Subsequently, taking advantage of newer technologies, DNA samples of 41 patients were analyzed by massive sequencing of a panel of genes related to haematological disorders including all the genes previously associated to telomere-related diseases (Additional file 1: Table S2). Out of these patients, 23 were diagnosed of DC/AA and 18 of PF.

**Table 1:**
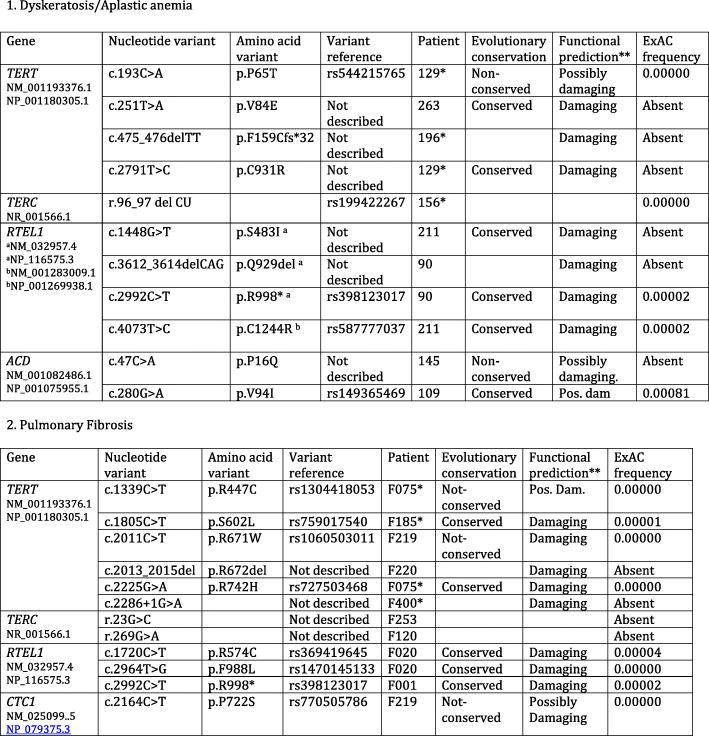
Mutations found in telomere-related genes

*Patients analyzed by exon amplification and Sanger sequencing

**The prediction shown is a summary of the ones made by the programs SIFT ensemble 66, Polyphen-2 v2.2.2, MutationAssessor, release 2, FATHMM v2.3, CADD v1.3 and dbscSNV1.1.

Nucleotide variants were further analysed when their frequency in the general population was lower than one in a thousand. The results obtained for genes previously related to telomere shortening are shown in Table 1. These studies allowed the identification of single nucleotide variants (SNVs) and indels at *TERT*, *TERC*, *RTEL1*, *ACD* and *CTC1* genes in heterozygosis. Histograms of the Sanger sequencing reaction confirming these variants are shown in Additional file 1: Figure S2.

**Fig. 1:**
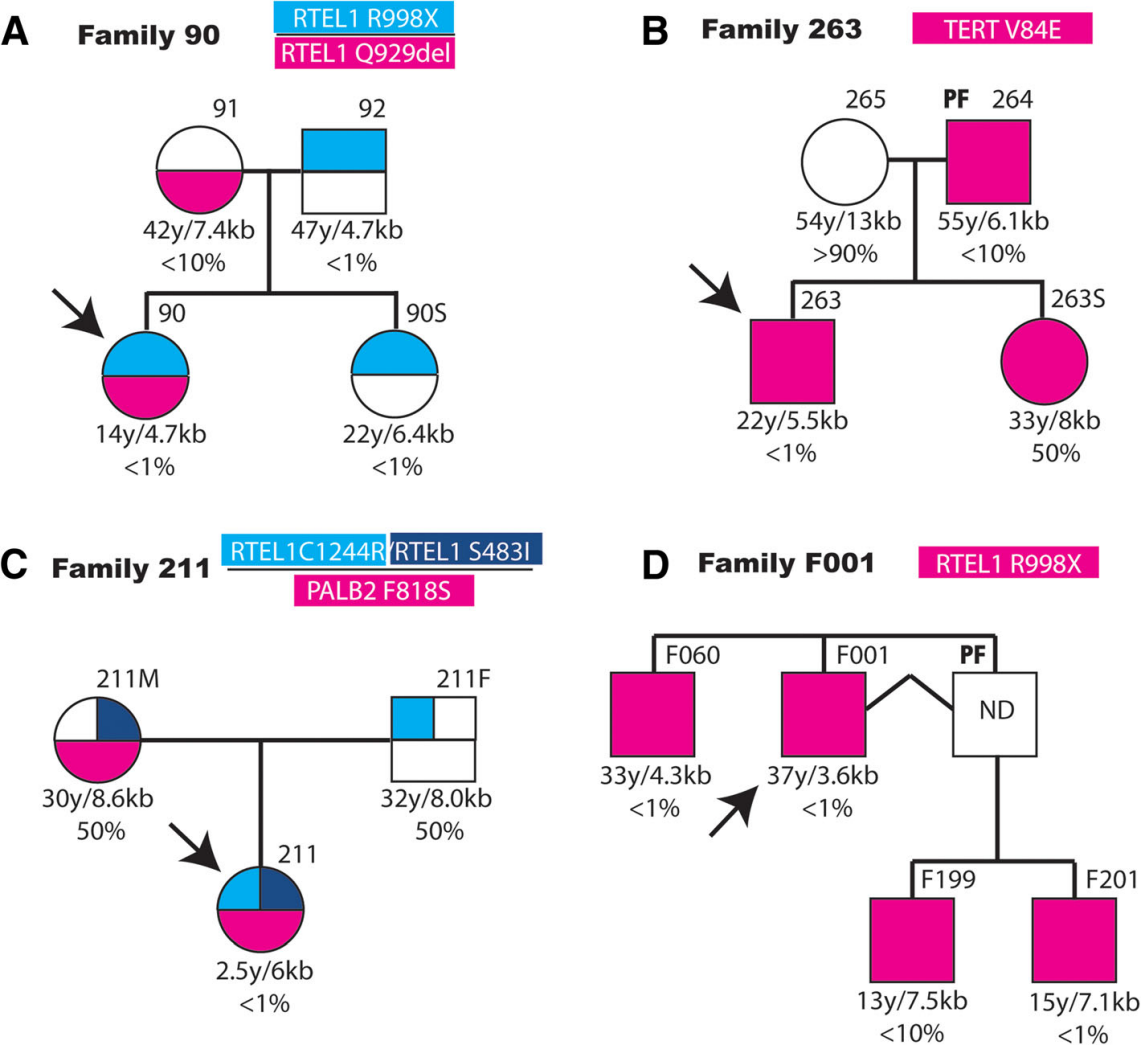
Familiar inheritance of variants in telomere-related genes in patients with aplastic anemia and pulmonary fibrosis. The family of four affected patients is represented in panels (**a**-**d**). The probands (indicated by arrows) of families (**a**-**c**) are affected of aplastic anemia and that of family (**d**) of pulmonary fibrosis. The number of the family and the variants analyzed are shown in the upper part of each diagram. In the cases where several variants were studied, they are shown in the heading in the same relative order and colour as in the diagrams. The presence of the variants is represented as colour-filled squares or circles and that of the presence of the reference allele as empty figures. The number assigned to each patient or relative is indicated in the upper, right part of each symbol. The age of each individual, the determined telomere size, in kb, and the corresponding percentage of the age-matched population are shown under each symbol. Relatives affected by pulmonary fibrosis are indicated in the upper left part of their symbol (PF). Patient F001 in panel (**d**) had a deceased identical twin brother as indicated by joining them with a broken line

*TERT* variants were found in eight patients (Table 1). AA patient 129 presented two missense variants, p.P65T and p.C931R. The p.P65T variant was inherited from the mother of the patient, which also presented short telomeres. The p.C931R variant was not present in the parents of the patient. AA patient 196 presented in heterozygosis a deletion of two nucleotides at positions c.475_476 that produced a frame shift mutation after the amino acid residue 158. One stop codon will be found 31 residues downstream, resulting in a 189 amino acids-long protein. This variant was inherited from the father of the patient, which presented PF. A son of the patient that also presented this variant, died before birth. AA patient 263 carried in heterozygosis the *TERT* p.V84E variant. His father also carries the variant in heterozygosis and presents PF and short telomeres. A sister that carries the variants is presently unaffected at the age of 33 years (Fig. 1).

PF patient F075 presented two missense variants at *TERT*, p.R447C and p.R742H. The variant p.R742H has been already reported [23] while the p.R447C variant is described for the first time. The mother and a brother of this patient died of PF. PF patient F185 presented the p.S602 L amino acid variant. PF patient F219 presented the p.R671W variant, reported to be pathogenic [24]. PF patient F220 presented an in-frame deletion at amino acids 671_672 that has not been previously described. A variant in a splicing consensus site was found in patient F400.

Variants in *TERC* were found in three patients (Table 1). AA patient 156 presented in heterozygosis a deletion of two bases (r.96_97delCU) that has been previously described [25]. The mother of the patient died with PF and liver cirrhosis, a brother carrying this variant also presented liver cirrhosis and short telomeres. However, a sister that carries the variant is presently healthy at the age of 40 years even if her telomeres are short. PF patient F120 presented the r.269G > A variant, that has not been previously reported. PF patient F253 presented the undescribed r.23G > C variant. PF patient F219 presented the p.P722S variant in *CTC1* that has not been previously described.

Four patients presented rare variants in *RTEL1* in heterozygosis (Table 1). AA patient 90 presented the p.R998* variant recently reported as pathogenic [26]. The father and one sister of the patient carried this variant, and presented short telomeres (Fig. 1). A representative Southern blot of telomeres including relatives of patient 90 is shown in Additional file 1: Figure S1. In addition, patient 90 and mother carried the p.Q929del variant, recently described [26]. AA patient 211 presented the new pS483I variant and the described p.C1244R variant [27]. In addition, patient 211 presented the amino acid variant p.F818S in *PALB2* protein, also involved in telomere maintenance, as will be discussed later. The *RTEL1* variant p.C1244R was inherited from the father and the *RTEL1* variant p.S483I and the *PALB2* variant from the mother and both parents presented average telomere length (Fig. 1). PF patient F001 presented the p.R998* pathogenic variant. This *RTEL1* variant was present in the three analyzed relatives of patient F001, all with short telomeres (Fig. 1). PF patient F020 presented the described p.F988 L [15] and the non-described p.R574C variants.

Nucleotide variants in *ACD*, coding for the TPP1 shelterin protein, were observed in two patients. AA patient 145 presented the p.P16Q amino acid variant and AA patient 109 the p.V94I variant, both in heterozygosis. The variant of patient 145 is inherited from the mother that reports several relatives affected of DC. Patients 109 and 145 presented chromosome 7 monosomy.

### Single nucleotide variants in genes related to DNA repair and homologous recombination

Massive sequencing identified a number of rare SNVs (frequency lower than one in a thousand) in genes coding for proteins involved in DNA repair by homologous recombination, as shown in Table 2. Among these genes are those coding for the protein kinases ATM (Ataxia-telangiectasia mutated) and ATR (Ataxia telangiectasia and Rad3-related), mutated in the genetic-instability syndromes Ataxia Telangiectasia and Seckel syndrome, respectively. Rare variants in these proteins were found in 35% of the DC/AA and 17% of the PF patients (Table 2). Fanconi Anemia is also due to cytogenetic instability, hypersensitivity to DNA crosslinking agents and defective DNA repair. This disease can be caused by mutation in genes that code for components of a protein complex involved in DNA repair. Rare variants in several of these genes (*FANCA*, *FANCD2*, *FANCF*, *FANCL*, *FANCM*, *FANCU*) were found in 22% of the DC/AA and PF patients (Table 2). The DNA- associated proteins BRCA1 and BRCA2 (Breast Cancer 1 and 2) also play an important role in DNA break repair and recombination in association with PALB2 (Partner and Localizer of BRCA2). Rare variants in the *BRCA1*, *BRCA2* or *PALB2* genes were found in 26% of the DC/AA and 17% of the PF patients (Table 2). RecQL4 is a DNA helicase (RecQ-like, type 4) involved in DNA damage repair and rare variants were found in 17 and 11% of DC/AA and PF patients, respectively. Variants in the gene coding for the structure-specific-endonuclease subunit SLX4, involved in DNA repair and replication, were found in 22% of the DC/AA and 6% of the PF patients (Table 2). Mutations in *SLX4* have been associated with Fanconi Anemia (group P). Finally, single rare variants were found in other genes coding for proteins involved in DNA repair such as *WRN*, *BLM*, *RAD51*, *RAD51C* and *NBN* in 9 and 17% of the DC/AA and PF patients, respectively. The variants found in these genes for each patient are shown in Table 3. Some of these variants are described for the first time as indicated (“Not described” in Table 3).

**Table 2:** Frequency of rare variants in genes coding for proteins involved in DNA-Repair

	Dyskeratosis/Anemia	Pulmonary Fibrosis
*ATM*/*ATR*	8/23 (35%)	3/18 (17%)
*BRCA1*, *BRCA2*, *PALB2*	6/23 (26%)	3/18 (17%)
*FANC-A*, *−D2*, *−F*, *−L*, *−M*, *−U*	5/23 (22%)	4/18 (22%)
*RECQL4*	4/23 (17%)	2/18 (11%)
*SLX4*	5/23 (22%)	1/18 (6%)
*WRN*, *BLM*, *NBN*, *RAD51*, *RAD51C*	2/23 (9%)	3/18 (17%)

**Table 3:**
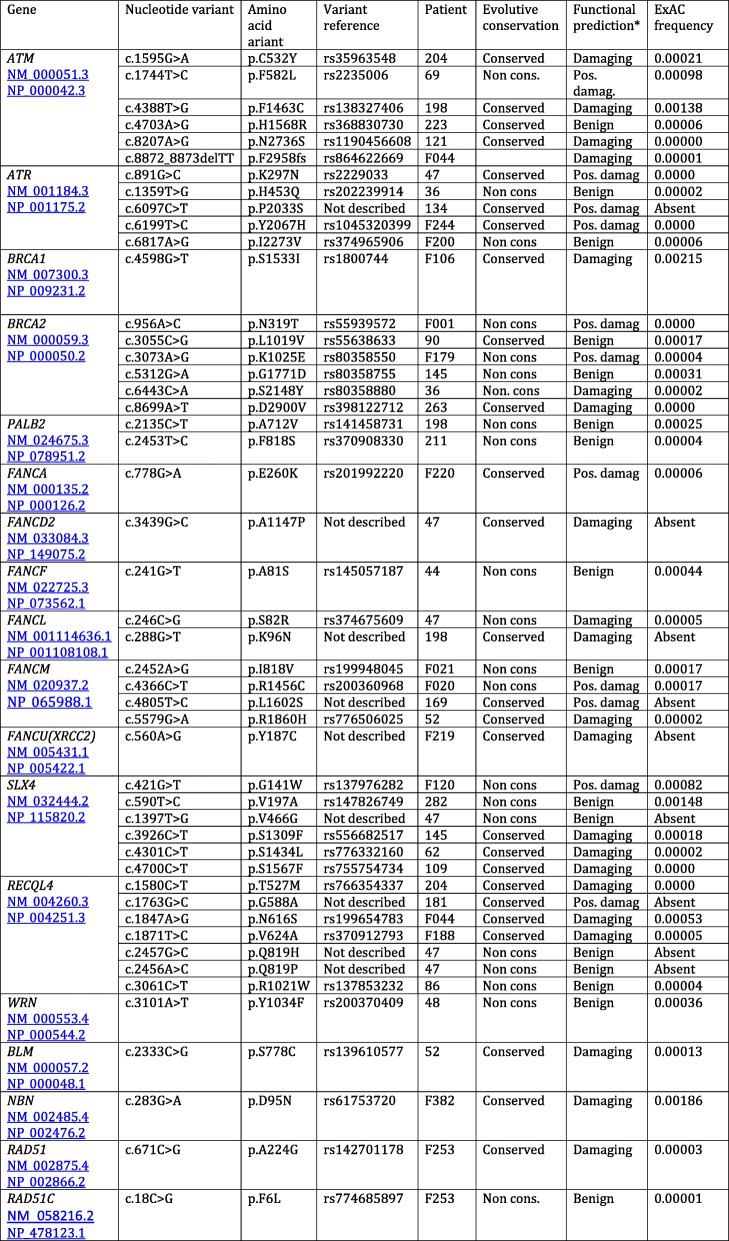
Variants in DNA repair genes

*The prediction shown is a summary of the ones made by the programs SIFT ensemble 66, Polyphen-2 v2.2.2, Mutation Assessor, release 2, FATHMM v2.3, CADD v1.3 and dbscSNV1.1.

The variants present in each patient and their clinical evolution from diagnosis are summarized in Table 4. Two patients with previously described DKC1 mutations, 26 and 36 [20], are included. This table includes some variants whose frequency is slightly higher than one in a thousand (indicated in bold letters). One of them is a variant in the gene *POT1* (pQ301H) present at a frequency of 0.002 in the ExAC database. The change is possibly damaging and is present in patient F106. A second variant in *NHP2* (p.R101Q) is present at a frequency of 0.002 at the ExAC database. The change is possibly damaging, and has been detected in three patients (F200, F120 and 223). Among the proteins involved in DNA repair, two patients (F20, 109) present the p.T287A variant at the *RAD51C* gene (frequency of 0.003 in the ExAC database). Other two patients (26 and 134) present the p.A492D variant at the *MRE11A* gene (frequency 0.002 in ExAC).

**Table 4:** Genes coding for proteins involved in telomere biology and DNA repair in which rare variants were found in the different patients and clinical evolution

Patient	Telomere biology genes	DNA repair genes	Clinical evolution
Pulmonary Fibrosis			
F001	*RTEL1*	*BRCA2*	Deceased
F010			Stable
F020	*RTEL1*	*FANCM*, ***RAD51C***	Lung transplant
F021		*FANCM*	Deceased
F025			Lung transplant
F044		*ATM*; *RECQL4*	Disease progressing
F075*	*TERT*	N.D.	Deceased
F106	***POT1***	*BRCA1*	Lung transplant
F120	*TERC*, ***NHP2***	*SLX4*	Deceased
F179		*BRCA2*	Disease progressing
F185*	*TERT*	N.D.	Deceased
F188		*RECQL4*	Disease progressing
F200	***NHP2***	*ATR*	Deceased
F213			Disease progressing
F219	*TERT*, *CTC1*, *RTEL1*	*FANCU*	Deceased
F220	*TERT*	*FANCA*	Lung transplant
F242			Lung transplant
F244		*ATR*	Disease progressing.
F253	*TERC* ***, CTC1***	*RAD51*, *RAD51C*	Deceased
F382		*NBN*	Disease progressing
F400*	*TERT*	N.D.	Waiting transplant
Dyskeratosis/Aplastic anemia			
26	*DKC1* ^Ref 19^	***Mre11A***	Deceased
36	*DKC1* ^Ref 19^	*ATR*, *BRCA2*	Disease progressing
44		*FANCF*	Stable
47		*ATR*, *FANCD2*, *FANCL*, *RECQL4*, *SLX4*	Recovered
48		*WRN*,	N.D.
52		*FANCM*, *BLM*	HSC transplant
62		*SLX4*	HSC transplant
69		*ATM*	Stable
86		*RECQL4*	Stable
90	*RTEL1*	*BRCA2*	HSC transplant
109	*ACD*	*SLX4*, ***Rad51C***	HSC transplant
121		*ATM*	HSC transplant
129*	*TERT*	N.D.	Deceased
134		*ATR*, ***Mre11A***	Stable
145	*ACD*	*BRCA2*, *SLX4*	Deceased
156*	*TERC*	N.D.	Deceased
169		*FANCM*	Deceased
181		*RECQL4*	Stable
196*	*TERT*	N.D.	Deceased
198		*ATM*, *FANCL*, *PALB2*, ***RAD50***	Disease progressing
204		*ATM*, *RECQL4*	Stable
205			Stable
211	*RTEL1*	*PALB2*	HSC transplant
223	***NHP2***	*ATM*	Stable
263	*TERT*, *RTEL1*	*BRCA2*, *SLX4*	HSC transplant
282		*SLX4*	N.D.

*N.D.* Not determined. Boldface: genes that present variants with a frequency between 0.001 and 0.003 in the ExAC database.

**Fig. 2:**
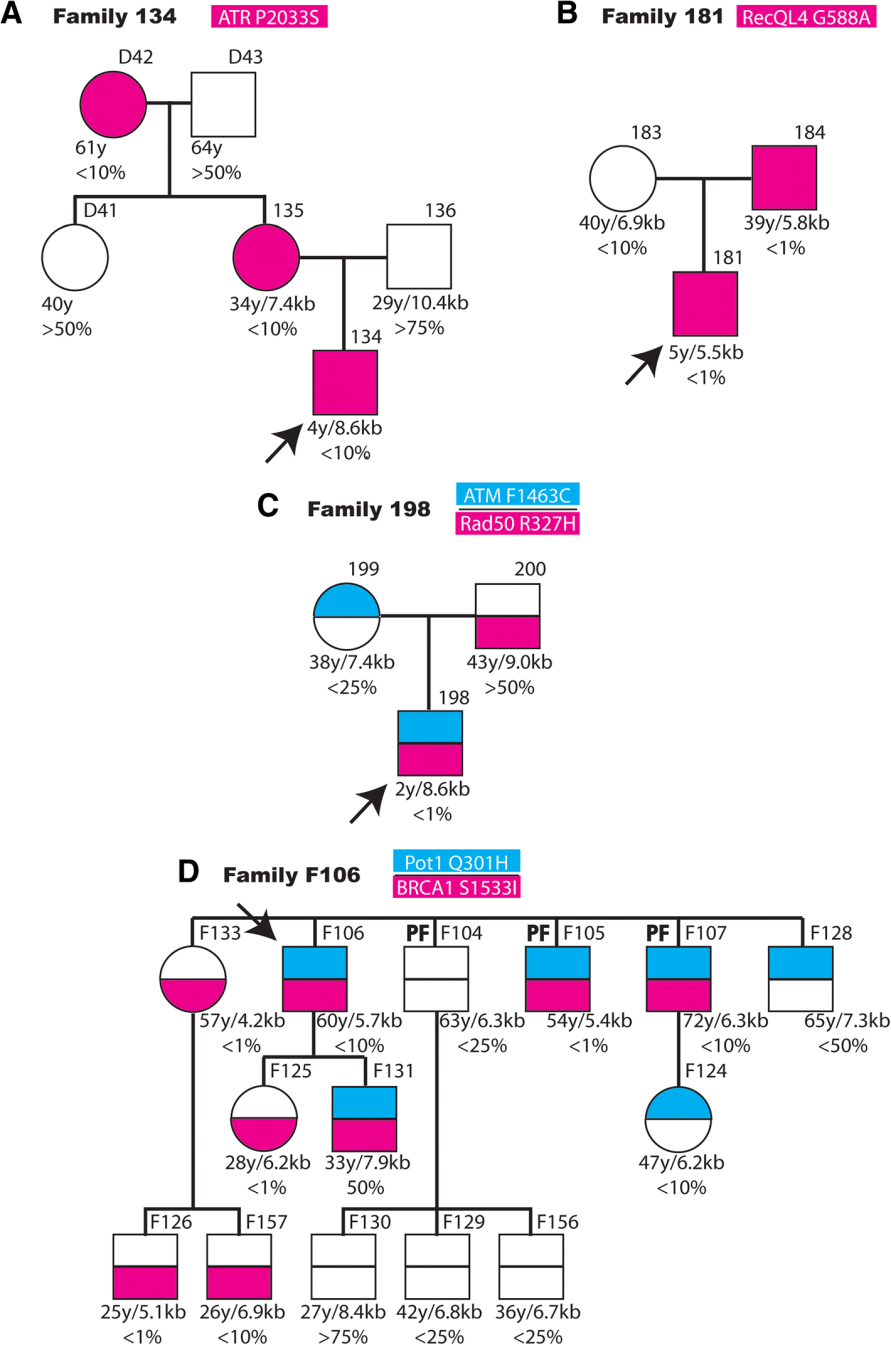
Familiar inheritance of variants in genes related to DNA repair and replication in patients with aplastic anemia and pulmonary fibrosis. Schematic representation of four families of patients (indicated by arrows) affected by aplastic anemia (**a**-**c**) or pulmonary fibrosis (**d**). The number of each family and the variants studied are shown in the upper part of each panel. The presence of the rare variant is indicated as coloured square or circles and the presence of the reference variant by open figures. In panels (**c** and **d**) where several variants were studied, their relative position in the diagrams and colour are indicated in the upper part. The number of each family member and its possible pulmonary fibrosis diagnosis (PF) is indicated in the upper part of each symbol. The age, telomere length (kb) and the corresponding percentage of the age-matched population are indicated in the lower part of each symbol

The possible correlation between the presence of rare variants in proteins involved in DNA repair or replication, telomere length and clinical manifestation was investigated in the family of several patients (Fig. 2). AA patient 134 carries one variant in the *ATR* gene not previously described. The variant is inherited from his mother and grandmother, both relatives have short telomeres. The father and maternal grandfather, that do not carry this variant, present an average telomere size.

AA patient 181 present a variant in *RECQL4* not described previously. This variant is present in the father with very short telomeres. The mother does not carry this variant and has longer telomeres even that below the average of the population (10%).

AA patient 198 presents a novel variant in *ATM* and a rare variant in *RAD50*. The *ATM* variant is inherited from the mother and the *RAD50* p.R850Q variant from the father (Fig. 2). The father present telomeres of average size and the mother of size bellow the average. However, the son presents relatively shorter telomeres than the parents.

The PF patient F106 carries a rare variant in *BRCA1* and a relatively rare variant in *POT1* (frequency 0.002). The patient has four brothers and three have been also diagnosed of PF. Two of them carry the same variants in *POT1* and *BRCA1* and short telomeres. A sister carries only the *BRCA1* variant and very short telomeres although is presently asymptomatic. Two of the brothers carry exclusively the *POT1* mutation and present longer telomeres (25–50% of the population) although one of them has developed pulmonary fibrosis. The patient has a daughter, which inherited the *BRCA1* variant and present very short telomeres. Two nephews and a niece carry the *BRCA1* variant and have short telomeres. One niece inherited the *POT1* change and presents short telomeres.

## Discussion

The genetic variants present in patients with symptoms of DC, AA or PF have been studied in a population of patients from Spanish hospitals. Heterozygous variants were found in genes previously related to telomeropathies in 17 patients, 8 diagnosed of DC/AA and 9 of PF (Table 1). Two variants in *ACD* were found in the only two AA patients that also presented chromosome 7 monosomy, previously associated to the presence of short telomeres in AA patients as a consequence of chromosomal instability [28].

**Fig. 3:**
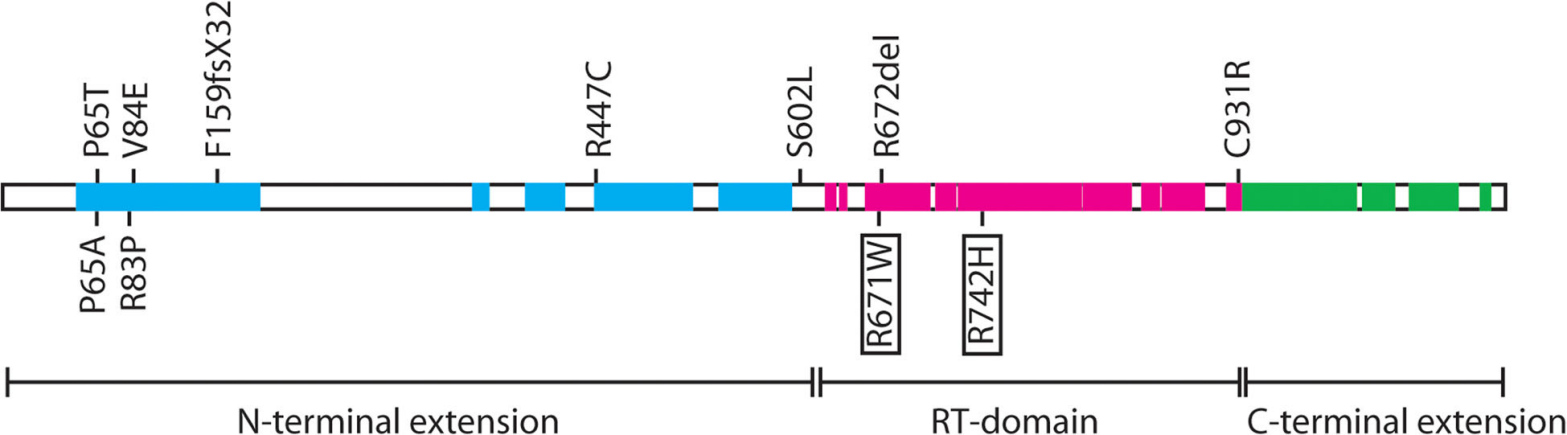
Schematic representation of the location of TERT variants on the conserved and functional domains of the protein. Conserved regions of TERT are represented as filled boxes and coloured according to their participation in the protein functional domains (shown in the lower part of the scheme). Regions of the N-terminal extension, involved in RNA binding, are coloured in blue. Regions of the reverse transcriptase domain (RT-domain) are shown in cyan and those of the C-terminal extension in green. The location of the new amino acid variants described in the article is shown in the upper part of the figure. Two previously described variants close to new variants (P65A and R83P) are shown in the lower part of the figure. The location of two variants found in this project and previously described as pathogenic are shown in the lower part inside boxes

Several of the variants found had been reported previously as pathogenic. The *TERT* variants p.R671W and p.R742H have been described associated to PF [23, 24]. The *TERC* variant r.96_97delCU was described in a DC patient transmitted with a dominant pattern of inheritance [25]. The *RTEL1* variants p.Q929del [26] pF988 L [29], p.R998* [26] and p.C1244R [27] were described in DC patients. The *RTEL1* p.R574C variant has been described as a clinical variant. The other variants found in this study have not been previously reported and according to the actual guidelines should be considered as variants of unknown significance (VUS). However, we speculate that some of them could be pathogenic because they are closely related to described pathogenic variants, as schematically shown in Fig. 3. This is the case of *TERT* p.P65T variants since the p.P65A variant has been found in patients of acute myeloblastic leukemia (AML) [26]. The *TERT* p.V84E variant is close to the p.R83P variant, found in patients with AA and myelodisplastic syndrome [30]. In addition, the V84 residue is conserved through evolution and the V > E change is considered as damaging. Other variants produced changes potentially damaging in conserved residues located in functional domains of the proteins like the *TERT* variants p.F159fsX32 and p.S602 L that affect residues located in the conserved N-terminal domain (Fig. 3). The p.F159fsX32 variant would produce a truncated protein and a situation of functional hemizygosis. The variants p.R672del and pC931R are located in the reverse transcriptase and C-terminal domains of *TERT*, respectively (Fig. 3). In the case of the *RTEL1* gene, the pS483I variant in a conserved residue is close to the p.P484L variant associated to PF [31].

The gene panel used for the analyses of the DNA of 41 patients by massive sequencing detected the presence of numerous variants in genes coding for proteins involved in DNA repair such as *ATM*, *ATR*, *BRCA1*, *BRCA2* and several genes of the FANC complex, mutated in Fanconi Anemia. Variants in genes coding for DNA helicases and nucleases involved in the resolution of DNA complex structures, such as *SLX4*, *RECQL4*, *WRN*, *BLM*, *RAD51*, *RAD51C* or *NBN* were also found. Variants in these genes have not been related to telomeropathies previously, with the exception of *SLX4* [32].

The proteins ATM and ATR play a signalling role in DNA double-strand break repair pathways and their continued activation can result in cell cycle arrest, replicative senescence or apoptotic cell death. However, specific interactions with shelterin protein, ATM/TRF2, and ATR/TRF1, inhibit their activation to preserve telomere structure. Therefore, it is conceivable that functional variants at ATM and ATR could affect telomere structure and stability. Indeed, cells of Ataxia telangiectasia patients with homozygous mutations in *ATM* present telomere shortening and increase frequency of chromosome end-to-end fusions (CEFs) [33].

BRCA1 and BRCA2 proteins also participate in DNA damage repair by homologous recombination and non-homologous end joining [34] and play a protective role at telomeres [35]. *BRCA1/2* mutations result in shortening of the telomere single-stranded overhang and increase telomere length variability [36]. *BRCA2* heterozygous cell lines present frequently CEFs [37]. Germline mutations in *BRCA1* and *BRCA2* are associated with greatly increased frequency of breast and ovarian cancers and telomere shortening was associated with genetic anticipation in hereditary breast cancer [38].

Proteins mutated in Fanconi Anemia (FA) patients are involved in repair of DNA-inter-strand crosslinks and have been also involved in telomere maintenance (reviewed by Sarkar and Liu [39]). FA patients are reported to present increased CEFs and overall shorter telomeres [40, 41].

Replication of telomeric DNA presents specific challenges due to structure of the DNA with unconventional regions including T loop and G quadruplexes and requires the participation of a large number of proteins (reviewed by Martinez and Blasco [6]). Among them are proteins with DNA helicase activity, like RTEL1, BLM, WRN or RecQL4, endonuclease complexes, like the one formed by SLX4, SLX1, MU881 and XPF and recombinases like Rad51 and Rad51C.

Mutations in these genes cause diseases with some symptoms resembling those of telomeopathies, as mentioned in the Background section. Many of these diseases show telomere fragility [42], telomere loss and/or increased CEFs [43]. These proteins are located to telomeres through interactions with TRF1 of TRF2 shelterin proteins [44–48] . In addition, TERT expression in cells isolated from patients of these diseases rescue some of their alterations [49, 50]. Variants in *SLX4* have been found with increased frequency in AA patients [32].

The existence of overlapping symptoms in diseases caused by mutations in genes coding for proteins involved in telomere-maintenance and in DNA-repair and replication proteins have been recently reviewed [18]. In this article we describe the presence of rare variants in many of these genes in patients that present short telomeres and telomeropathies such as DC, AA and PF. We consider the possibility that the presence of one or more variants in these genes in heterozygosis, some times in addition to variants in telomere maintenance genes, could have a cumulative pathogenic effect. This association could be extensive to some more frequent variants in genes involved in primary telomeropathies, as those observed in *NHP2* or *POT1*. This possible pathogenic association need to be confirmed by functional studies and analyzing more extensive cohorts of patients.

The small number of patients analyzed prevents a statistical analysis of the possible genotype-phenotype correlation. There seems to be, however poor outcome in patients that carry variants in genes related to telomere biology, as shown in Table 4. For example, nine AA patients carried variants in telomere genes and five of them died in the course of the study and the other four required hematopoietic stem cell transplantation (HSCT). In contrast, only one patient died and three required HSCT of the sixteen that presented short telomeres but not rare variants in telomere related genes. Similar evolution was observed in PF patients. Nine carried mutations in telomere genes and four died and four required lung transplant. Of the 12 patients without mutations in these genes, two died and three required lung transplant. In particular, the two patients that carried two *TERT* variants evolved very quickly and both died in a few months after the diagnosis of the disease. One of these patients was diagnosed of AA and the other of PF. Among the patients with worst evolution without mutations in telomerase genes, three presented variants in *FANCM* (two died and one required HSCT). However, the analysis of a larger cohort of patients will be required to confirm or discard these proposals.

## Conclusions

Using massive sequencing of a panel of genes and Sanger sequencing of selected exons we identified novel variants in telomere biology genes in a series of patients of DC, AA and PF form Spanish hospitals. In additions we describe the existence of frequent rare variants in genes involved in DNA damage response and the resolution of complex DNA structures in this population of patients. Achieving a molecular diagnosis from patients of these telomere biology diseases would be important for accurate genetic counselling and effective treatment of the patients.

## Additional file

Additional file 1: Supplemental methods. **Table S1A.** Clinical characteristics of the Dyskeratosis congenita, Aplastic anemia patients. **Table S1B.** Clinical characteristics of Pulmonary fibrosis patients. **Table S2.** Genes included in the panel used for massive sequencing. **Table S3.** Description of the control population used for the estimation of normal mean, telomere length and percentile for each age-range. **Figure S1.** Representative Southern blot used for telomere length determination. **Figure S2.** Histograms of the sequences obtained by Sanger sequencing of the regions of genes related to telomere biology that presented SNVs or indels. (DOC 3474 kb)
