# Supplementary material for: Genetic analyses of aplastic anemia and idiopathic pulmonary fibrosis patients with short telomeres, possible implication of DNA-repair genes
Source: Orphanet J Rare Dis. 2019 Apr 17;14:82. doi: 10.1186/s13023-019-1046-0 (PMC6471801; doi:10.1186/s13023-019-1046-0)
Supplement: Supplementary file 1 — Supplemental methods. Table S1A. Clinical characteristics of the Dyskeratosis congenita, Aplastic anemia patients. Table S1B. Clinical characteristics of Pulmonary fibrosis patients. Table S2. Genes included in the panel used for massive sequencing. Table S3. Description of the control population used for the estimation of normal mean, telomere length and percentile for each age-range. Figure S1. Representative Southern blot used for telomere length determination. Figure S2. Histograms of the sequences obtained by Sanger sequencing of the regions of genes related to telomere biology that presented SNVs or indels. (DOC 3474 kb) [file 13023_2019_1046_MOESM1_ESM.doc]

Supplemental Materials

Supplemental Methods.

Genetic Analyses

The existence of sequence variations in the TERT, TERC and DKC1 genes was determined in a group of patients by PCR amplification of exons, high resolution melting analyses (HRM) and sequencing of the exons with possible nucleotide variants as previously described (1). Twenty nanograms of patient’s DNA were used for PCR amplification using the HotShot Diamond^TM^ Master Mix (Durvitz). HRM was performed on a Light Scanner^T^ system (Idaho Technology Inc.) and analyzed using the Lightscanner 2v software (Idaho). Samples that presented differences in the HRM profile were treated with Exosap (USB-Affymetric) and sequenced in both strands using an ABI 3130XL Genetic Analyzer (Applied Biosystems). All the variations found were confirmed on a second PCR product.

Genetic analysis of most of the patients was made by massive parallel sequencing using a panel of genes previously related to haematological disorders, as shown in Table S1. (MBFSv1.1 panel) This panel was designed with NimbleDesign software ([https://design.nimblegen.com](https://design.nimblegen.com/), Roche NimbleGen, Inc. USA): HG19 NCBI Build 37.1/GRCh37, target bases covered 99.6% and size 370,730Kb. For each sample, paired-end libraries were created according to the standard protocols using the KAPA HTP Library Preparation Kit for Illumina® platforms, SeqCap EZ Library SR (Roche NimbleGen, Inc. USA) and NEXTflex-96 Pre Capture Combo Kit (Bioo Scientific) for indexing. Captured sample DNA was sequenced on a NextSeq 500 instrument (Illumina, Inc. USA) using a high cartridge v2, according to the standard operating protocol.

Bioinformatic analysis was carried out by the Clinical Bioinformatics Unit of INGEMM. Software: trimmomatic-0.32, bowtie2-align version 2.1.0, picard-tools 1.106, samtools Version.0.1.19-44428cd, bedtools v2.18.1, GenomeAnalysisTK version 3.3-0. Data Bases: dbNSFP version 3.0, dbSNP v138, ClinVar date 20140703, SnpE 4.1l, Exac r0.3, SIFT ensembl 66, Polyphen-2 v2.2.2, MutationAssessor, release 2, FATHMM, v2.3, CADD, v1.3 and dbscSNV1.1.

Telomere length determination

Telomere length was determined by two different methods, Southern blots of enzymatically digested DNA and quantitative PCR. Both methods have been previously used for determination of telomere length from patients with inherited bone marrow failure (2). Southern blot analyses was used for DNA isolated from peripheral blood mononuclear cells (PBMCs) obtained by centrifugation in Ficoll-Page gradients. DNA was extracted from these cells as previously described (1). Telomere length was determined using the TeloTAGGG Telomere Length Assay (Roche) using 1 μg of genomic DNA. A representative Southern blot is presented in Figure S1.

Quantitative PCR was used to determine telomere length from DNA isolated from buccal cells collected using oral swabs (cytobrush). DNA was extracted using a DNA isolation kit (Isohelix, Cell Projects Ltd) (3). The relative telomere length (TL) was measured by using an optimized version of the quantitative PCR method previously described by Cawthon (4) that determines the ratio of telomere (T) repeat copy number to single-copy (S) gene (*36B4*) copy number (called T/S ratio) in experimental samples as compared with a reference DNA sample. A Z-score was obtained that compare the T/S ration in each individual with that of age-matched controls. Telomere shortening was considered when the Z-score was below the 10^th^ percentile. Using the telomere length of the reference MCF7 DNA (4,8 Kb), the T/S values obtained by qPCR were transformed to Kb.

Telomere qPCRs and single-copy gene (*36B4 gene)* qPCRs were performed in separate wells using the following primers: TelF (5′-CGG TTT GTT TGG GTT TGG GTT TGG GTT TGG GTT TGG GTT-3′) and TelR (5′-GGC TTG CCT TAC CCT TAC CCT TAC CCT TAC CCT TAC CCT-3′), used at final concentration of 900 nM each; 36B4F (5′-CAG CAA GTG GGA AGG TGT AAT CC-3′) and 36B4R (5′-CCC ATT CTA TCA TCA ACG GGT ACA A-3′), used at final concentration of 300nM each. Each 10µl amplification reaction volume contained 1x Power SYBR Green PCR Master mix (Applied Biosystems) and 4.5 ng of genomic DNA samples. Tubes containing 24, 6, 1.5 and 0.375 ng of a reference DNA (genomic DNA from human cell line MCF-7) were included in each run to allow the quantification of the samples relative to this reference DNA by the standard curve method. The R^2^ for each standard curve was ≥ 0.99 and the acceptable SD set at 0.2 for the Ct values. For inter-assays variability, a calibrator DNA sample was also included in each run. Each sample was run in triplicates. qPCRs were carried out on a Eco Real-Time PCR System (Illumina).

PCR cycling conditions for the telomere amplification were 95°C for 10 min, followed by 40 cycles of 95°C for 15 sec, 58°C for 30 sec and 72 °C for 30 sec, and for the *36B4* amplification: 95°C for 10 min, followed by 40 cycles of 95°C for 15 sec and 58°C for 1 min. Melting curve analysis was carried out at the end of each PCR experiment.

Control Population: DNA samples of healthy gender and age matched-controls. Exclusion criteria: subjects with cancer, diabetes, hematologic, respiratory, or hepatic diseases were excluded of our control group. The main characteristics of control populations are shown in Table S3. The table shows separately the age and gender distribution of the control populations used for the telomere length analysis of Pulmonary Fibrosis and Dyskeratosis/Anemia cohorts.

References

1. Carrillo J, Martinez P, Solera J, Moratilla C, Gonzalez A, Manguan-Garcia C, et al. High resolution melting analysis for the identification of novel mutations in DKC1 and TERT genes in patients with dyskeratosis congenita. Blood Cells Mol Dis. 2012 Oct 15-Dec 15;49(3-4):140-6.

2. Gadalla SM, Cawthon R, Giri N, Alter BP, Savage SA. Telomere length in blood, buccal cells, and fibroblasts from patients with inherited bone marrow failure syndromes. Aging (Albany NY). 2010 Nov;2(11):867-74.

3. McMichael GL, Gibson CS, O'Callaghan ME, Goldwater PN, Dekker GA, Haan EA, et al. DNA from buccal swabs suitable for high-throughput SNP multiplex analysis. J Biomol Tech. 2009 Dec;20(5):232-5.

4. Cawthon RM. Telomere measurements by quantitative PCR. Nucleic Acids Res. 2002 May 15;30(10):e47.

Table S1A. Clinical characteristics of the Dyskeratosis congenita, Aplastic anemia patients

| Patient Number | Sex | Age (years) | PBMC’s Telomere length (% aged population) | Clinical manifestations |
| --- | --- | --- | --- | --- |
| 26 | Male | 2 | 5.2 (<1%) | oral leukoplakia,  esophageal stenosis |
| 36 | Male | 32 | 5 (<1%) | pulmonary fibrosis,  Bone marrow failure |
| 44 | Female | 13 | 7.4 (<10%) | Bone marrow failure |
| 47 | Male | 10 | 8 (<10%) | Bone marrow failure. |
| 48 | Male | 9 | 8 (<10%) | interstitial lymphoid pneumonitis |
| 52 | Male | 2 | 8.6 (<1%) | Bone marrow failure, hepatosplenomegaly, increased transaminases |
| 62 | Male | 7 | 7.4 (<1%) | Bone marrow failure |
| 69 | Male | 15 | 6.8 (<10%) | Thrombocytopenia |
| 86 | Female | 4 | 8.4 (<10%) | Growth and  mental retardation,  bone marrow failure |
| 90 | Female | 14 | 4.7 (<1%) | Medular hypoplasia, eczematous skin lesions |
| 109 | Male | 0.5 | 8.6 (<1%) | Immunodeficiency, myelodysplastic syndrome, chromosome 7 monosomy |
| 121 | Male | 8 | 6.7 (<1%) | Refractory cytopenia |
| 129 * | Male | 39 | 5.4 (<1%) | Bone marrow failure, thrombocytopenia, interstitial pneumopathy |
| 134 | Male | 4 | 8.6 (<10%) | Poikiloderma  bone marrow failure |
| 145 | Male | 1 | 11.3 (<10%) | Pancytopenia, bone marrow failure chromosome 7 monosomy |
| 156 * | Male | 36 | 3.9  (<1%) | Bone marrow failure, pancytopenia, hepatic cirrhosis |
| 169 | Female | 0.4 | 11 (<10%) | Bone marrow failure |
| 181 | Male | 5 | 5 (<1%) | Ulcerative colitis, cerebellar atrophy, mental retardation, hypogammaglobulinemia |
| 196 * | Female | 35 | 6.1 (<1%) | Skin pigmentation, slight nail dystrophy, oral cancer, spontaneous abortions |
| 198 | Male | 2 | 8.6 (<1%) | Bone marrow failure exocrine pancreatic insufficiency, scanty hair, growth retardation |
| 204 | Male | 31 | 5 (<1%) | Bone marrow failure |
| 205 | Male | 7 | 7.2 (<1%) | Bone marrow failure |
| 211 | Female | 2.5 | 6 (<1%) | Uterine growth retardation, thrombocytopenia, bone marrow failure, XXX Trisomy, Cerebral palsy |
| 223 | Female | 2 | 8.6 (<1%) | Bone marrow failure |
| 263 | Male | 22 | 5.5 (<1%) | Neutropenia, thrombocytopenia, exudative retinopathy |
| 282 | Male | 15 | 7.4 (<10%) | Bone marrow failure |

* These samples have not been analyzed by massive sequencing

Table S1B. Clinical characteristics of Pulmonary fibrosis patients

| Patient Number | Sex | Age (years) | Buccal cells’ Telomere length (% aged population) | Clinical manifestations |
| --- | --- | --- | --- | --- |
| F001 | Male | 37 | 3.6 (<1%) | Familial Pulmonary Fibrosis |
| F010 | Male | 71 | 5.6 (<1%) | Familial Pulmonary Fibrosis |
| F020 | Male | 49 | 6.0(<10%) | Familial Pulmonary Fibrosis |
| F021 | Male | 75 | 5.2 (<1%) | Familial Pulmonary Fibrosis |
| F025 | Female | 53 | 5.8 (<10%) | Familial Pulmonary Fibrosis |
| F044 | Male | 62 | 4.7 (<1%) | Familial Pulmonary Fibrosis dendriform pulmonary ossification |
| F075 * | Female | 48 | 6.1 (<10%) | Pulmonary Fibrosis, |
| F106 | Male | 60 | 5.7 (<10%) | Familial Pulmonary Fibrosis |
| F120 | Female | 57 | 6.1 (<10%) | Familial Pulmonary Fibrosis, |
| F179 | Male | 59 | 5.1 (<1%) | Familial Pulmonary Fibrosis, Emphysema |
| F185 * | Male | 76 | 6.0 (<10%) | Pulmonary Fibrosis |
| F188 | Male | 49 | 3.8 (<1%) | Familial Pulmonary Fibrosis |
| F200 | Male | 67 | 5.4 (<10%) | Familial Pulmonary Fibrosis, Follicular lymphoma, bone marrow and skin affectation |
| F213 | Male | 46 | 3.7 (<1%) | Familial Pulmonary Fibrosis |
| F219 | Female | 68 | 4.3 (<1%) | Familial Pulmonary Fibrosis |
| F220 | Male | 58 | 5.3 (<1%) | Familial Pulmonary Fibrosis |
| F242 | Female | 48 | 6.2 (<10%) | Pulmonary Fibrosis, Emphysema |
| F244 | Male | 71 | 4.3 (<1%) | Pulmonary Fibrosis, Emphysema |
| F253 | Male | 51 | 2.7 (<1%) | Familial Pulmonary Fibrosis, Thrombocytopenia, cutaneous condition |
| F382 | Male | 63 | 5.1 (<10%) | Pulmonary Fibrosis, Emphysema |
| F400 * | Male | 57 | 5.7 (<10%) | Familial Pulmonary Fibrosis |

* These samples have not been analyzed by massive sequencing

Table S2. Genes included in the panel used for massive sequencing

| Telomere  Biology | Other genes and single nucleotide polymorphisms | | | | | | |
| --- | --- | --- | --- | --- | --- | --- | --- |
| ACD | ABCB7 | CSF3R | G6PC3 | MASTL | RPL15 | rs309557 | SRP72 |
| CTC1 | ADA | CXCR2 | GATA1 | MPL | RPL19 | rs3182911 | SRY |
| DKC1 | ADAMTS13 | CXCR4 | GATA2 | MRE11A | RPL26 | rs3736782 | STEAP3 |
| NHP2 | AK2 | CYCS | GFI1 | MYH9 | RPL27 | rs3744877 | TAZ |
| NOP10 | ALAS2 | DDX11 | GLRX5 | MYO5A | RPL31 | rs3749877 | TCIRG1 |
| PARN | ANKRD26 | DDX41 | GP1BA | NBEAL2 | RPL35A | rs4526148 | TRAF2 |
| POT1 | AP3B1 | DNA2 | GP1BB | NBN | RPL5 | rs514084 | TRAF3 |
| RTEL1 | ATM | DNM2 | GP9 | NFKB2 | RPS10 | rs532625 | TRAF6 |
| TERC | ATR | DTNBP1 | HAX1 | NHEJ1 | RPS14 | rs6596422 | TRNT1 |
| TERT | BACH1 | EFNB1 | HOXA11 | NIN | RPS17 | rs6686 | TSR2 |
| TINF2 | BLM | EIF2AK3 | HPS1 | PALB2 | RPS19 | rs6894087 | TUBB1 |
| WRAP53 | BLOC1S3 | ELANE | HPS3 | PAX5 | RPS24 | rs7808823 | UBE2T |
|  | BRCA1 | ERCC4 | HPS4 | PJA1 | RPS26 | rs8025851 | USB1 |
|  | BRCA2 | ERCC6L2 | HPS5 | PLDN | RPS27 | rs9284174 | VPS13B |
|  | C15ORF41 | ESCO2 | HPS6 | PUS1 | RPS28 | rs9302885 | VPS45 |
|  | CBL | ETV6 | IKBKB | RAB27A | RPS29 | rs9958735 | WAS |
|  | CD40 | FANCA | IL2RG | RAD50 | RPS7 | RUNX1 | WIPF1 |
|  | CDAN1 | FANCB | ITGA2B | RAD51 | rs1035705 | SBDS | WRN |
|  | CDY1 | FANCC | ITGB3 | RAD51C | rs1048479 | SBF2 | XRCC2 |
|  | CEBPA | FANCD2 | JAGN1 | RBBP8 | rs1249950 | SEC23B | ZFY |
|  | CENPJ | FANCE | KLF1 | RBM8A | rs2074262 | SLC19A2 |  |
|  | CEP152 | FANCF | LAMTOR2 | RECQL4 | rs2241190 | SLC25A38 |  |
|  | CEP63 | FANCG | LIG4 | REL | rs2296035 | SLC37A4 |  |
|  | chr3:169482398-169482848 | FANCI | LTBR | RELB | rs2303611 | SLX4 |  |
|  | chr9:35657748-35658015 | FANCL | LYST | RNF168 | rs2414865 | SMARCAL1 |  |
|  | CLPB | FANCM | MAP3K14 | RPL11 | rs28728105 | SPIN2A |  |

|  |
| --- |

Table S3. **Age and Gender distribution of the control populations**

| **Control Population of Pulmonary Fibrosis Cohort**  (Buccal cell telomere length analyzed by qPCR) | | | |
| --- | --- | --- | --- |
| Age distribution  (n= 171 subjects) | Mean age  (years) | Gender distribution  (n / % females) | Telomere Length (Kb)  (mean ±SD) |
| 30-39 years: n=52 | 34.5 | 37 / 71.1 | 8.0 ± 1.04 |
| 40-49 years: n=39 | 44 | 27 / 69.2 | 7.9 ± 1.19 |
| 50-59 years: n=31 | 55 | 22 / 70.9 | 7.5 ± 1.05 |
| 60-69 years: n=34 | 63.5 | 26 / 76.4 | 7.4 ± 1.46 |
| 70-79 years: n=15 | 74.5 | 9 / 60 | 7.5 ± 0.95 |
| **Control Population of Dyskeratosis/Anemia Cohort**  (PBMCs telomere length analyzed by Southern blot) | | | |
| Age distribution  (n= 79 subjects) | Mean age  (years) | Gender distribution  (n / % females) | Telomere Length (Kb)  (mean ±SD) |
| 0-5 years: n=17 | 2 | 8 / 47 | 11.4 ± 1.8 |
| 6-10 years: n=12 | 7.7 | 3 / 25 | 9.9 ± 2.0 |
| 11-20 years: n=10 | 14.5 | 3 / 30 | 9.3 ± 1.3 |
| 21-30 years: n=6 | 25.5 | 3 /50 | 9.2 ± 1.5 |
| 31-40 years: n=20 | 35.6 | 13 / 65 | 8.8 ± 1.8 |
| 41-50 years: n=14 | 43.9 | 6 / 42.8 | 8.9 ± 1.6 |


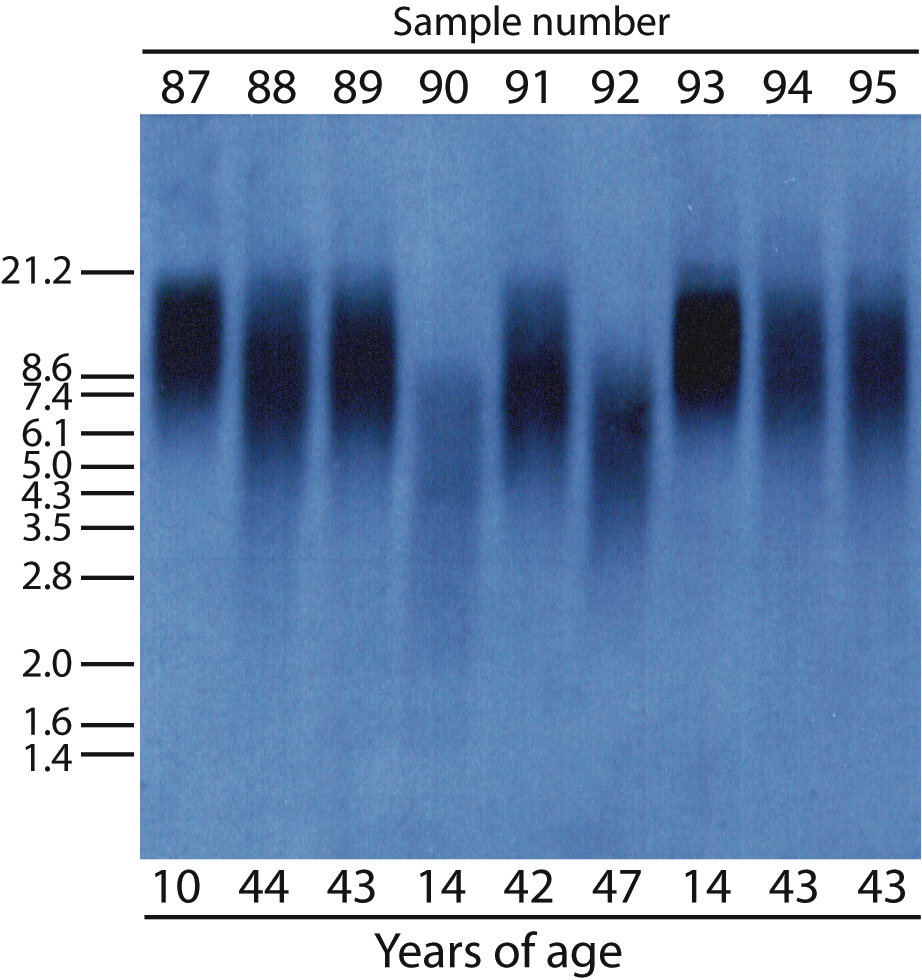


Figure S1. Representative Sothern blot used for telomere length determination.

DNA was isolated from peripheral blood mononuclear cells (PBMCs) and telomere length determined using the TeloTAGGG Telomere Length Assay (Roche). The number of each sample is shown in the upper part of the figure while the age of the corresponding patient or relative is shown in the lower part. The migration of molecular size markers, in Kb, is shown in the left column. Samples 87-89 and 93-95 correspond to two patients that did not present telomere shortening (87, 93), their mothers (89,94) and fathers (88,95). Sample 90 corresponds to a patient with significant telomere shortening (<1% of the age-mated control population), its father (92) that also had short telomeres (<1%) and mother with larger telomeres (<10%).


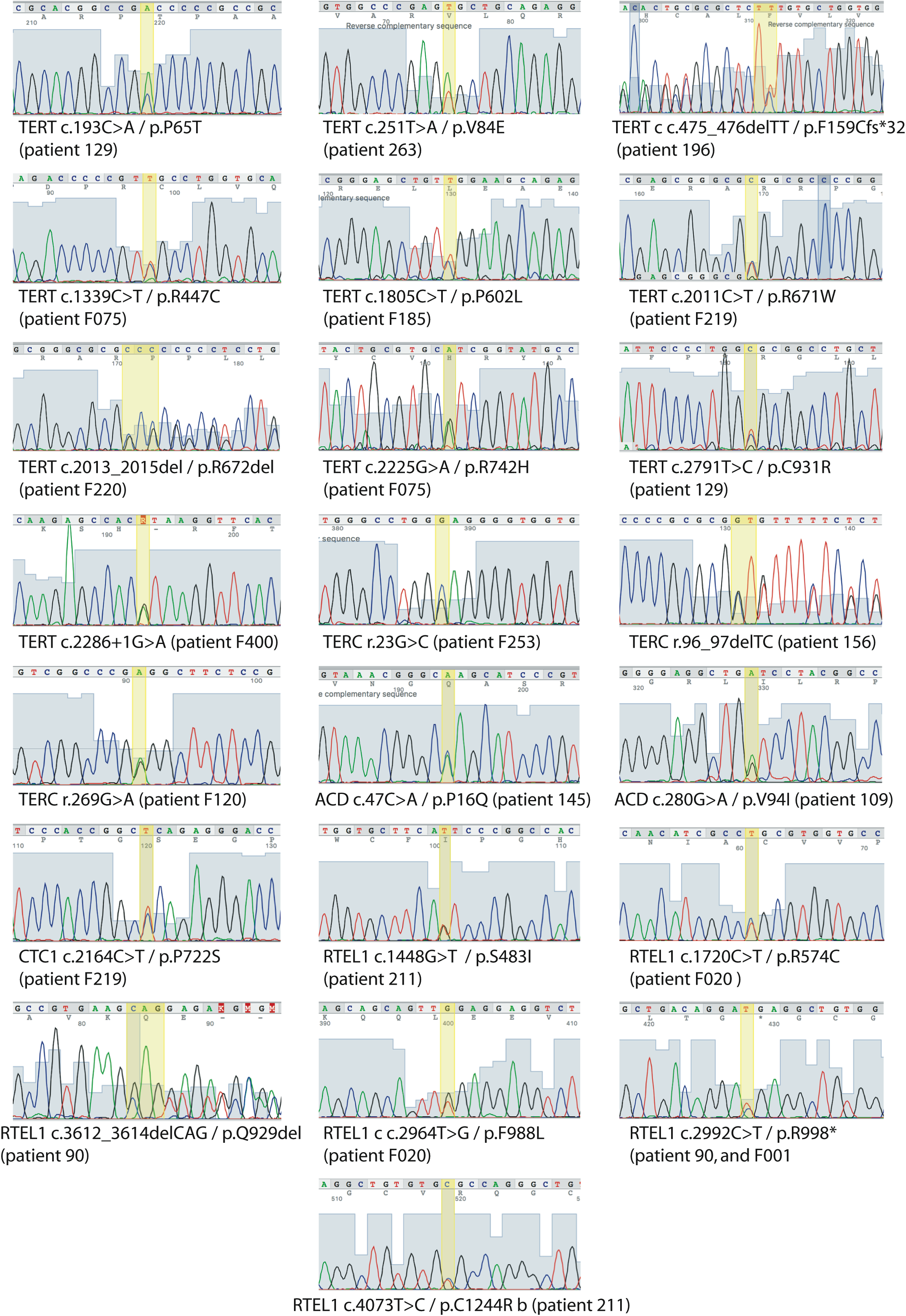


Figure S2. Sanger sequencing histograms of the regions of genes related to telomere biology that presented SNVs or indels.

Exons containing the analyzed single nucleotide variants (SNVs) or indels were amplified by PCR and the products obtained sequenced by the Sanger’s method. Histograms of the sequence of selected regions containing the variants are shown. The nucleotide sequence is shown in the upper part of the histogram and heterozygous positions are highlighted in yellow. The gene analyzed, nucleotide variant, amino acid variant and the number of each patient are shown in the lower part of the histograms.
